# The use of urinary proteomics in the assessment of suitability of mouse models for ageing

**DOI:** 10.1371/journal.pone.0166875

**Published:** 2017-02-15

**Authors:** Esther Nkuipou-Kenfack, Joost P. Schanstra, Seerat Bajwa, Martin Pejchinovski, Claire Vinel, Cédric Dray, Philippe Valet, Jean-Loup Bascands, Antonia Vlahou, Thomas Koeck, Melanie Borries, Hauke Busch, Wibke Bechtel-Walz, Tobias B. Huber, Karl L. Rudolph, Andreas Pich, Harald Mischak, Petra Zürbig

**Affiliations:** 1 Mosaiques Diagnostics GmbH, Hannover, Germany; 2 Hannover Medical School, Core Facility Proteomics, Hannover, Germany; 3 Institut National de la Santé et de la Recherche Médicale (INSERM), U1048, Institut of Cardiovascular and Metabolic Disease, Toulouse, France; 4 Université Toulouse III Paul-Sabatier, Toulouse, France; 5 Leibniz Institute of Age Research, Fritz Lipmann Institute, Jena, Germany; 6 Inserm-UMR Diabète athérothrombose Thérapies Réunion Océan Indien (DéTROI), plateforme CYROI, Sainte-Clotilde, France; 7 Biotechnology Division, Biomedical Research Foundation Academy of Athens, Athens, Greece; 8 Systems Biology of the Cellular Microenvironment Group, Institute of Molecular Medicine and Cell Research, Albert-Ludwigs-University, Freiburg im Breisgau, Germany; 9 German Cancer Consortium (DKTK), Freiburg and German Cancer Research Center (DKFZ), Heidelberg, Germany; 10 University Hospital Freiburg, Freiburg, Germany; 11 Department of Medicine IV, Faculty of Medicine, Medical Center, University of Freiburg, Freiburg, Germany; 12 BIOSS Center for Biological Signalling Studies, Albert-Ludwigs-University Freiburg, Freiburg, Germany; 13 Center for Systems Biology (ZBSA) and Freiburg Institute for Advanced Studies (FRIAS), Albert-Ludwigs-University Freiburg, Freiburg, Germany; 14 BHF Glasgow Cardiovascular Research Centre, University of Glasgow, Glasgow, United Kingdom; Cedars-Sinai Medical Center, UNITED STATES

## Abstract

Ageing is a complex process characterised by a systemic and progressive deterioration of biological functions. As ageing is associated with an increased prevalence of age-related chronic disorders, understanding its underlying molecular mechanisms can pave the way for therapeutic interventions and managing complications. Animal models such as mice are commonly used in ageing research as they have a shorter lifespan in comparison to humans and are also genetically close to humans. To assess the translatability of mouse ageing to human ageing, the urinary proteome in 89 wild-type (C57BL/6) mice aged between 8–96 weeks was investigated using capillary electrophoresis coupled to mass spectrometry (CE-MS). Using age as a continuous variable, 295 peptides significantly correlated with age in mice were identified. To investigate the relevance of using mouse models in human ageing studies, a comparison was performed with a previous correlation analysis using 1227 healthy subjects. In mice and humans, a decrease in urinary excretion of fibrillar collagens and an increase of uromodulin fragments was observed with advanced age. Of the 295 peptides correlating with age, 49 had a strong homology to the respective human age-related peptides. These ortholog peptides including several collagen (N = 44) and uromodulin (N = 5) fragments were used to generate an ageing classifier that was able to discriminate the age among both wild-type mice and healthy subjects. Additionally, the ageing classifier depicted that telomerase knock-out mice were older than their chronological age. Hence, with a focus on ortholog urinary peptides mouse ageing can be translated to human ageing.

## Introduction

During a lifetime, a number of molecular and cellular insults accumulate and lead to ageing [1]. Ageing is therefore a complex process characterised by a systemic and progressive deterioration of biological functions, leading to impaired tissue function thus increasing the likelihood of death. The burden caused by age-related diseases is prominent and prone to increase over the years. As life expectancy increases, improving health in the elderly population will be pivotal in dealing with subsequent enormous socio-economic challenges as a consequence of this improved longevity [2]. There is therefore an urgency to develop intervention strategies that will improve management of co-morbidities associated with ageing. Management of complications associated with ageing can firstly be accomplished by understanding molecular mechanisms associated with healthy ageing.

In ageing research, human studies are rare due to limiting factors mainly pertaining to the challenge in obtaining tissue samples from apparently healthy subjects [3]. As a result, animal models including mouse models have mostly been used due to obvious factors including shorter life span and the ease of obtaining samples in comparison to humans. A major concern of using animal models is the ability (or the lack thereof) to translate results to humans [4]. We have previously reported the benefit of using urinary proteome analysis in the screening of suitable animal models for human diseases [5,6]

In the present study our aim was to investigate if findings in ageing research using mouse models can be translated to humans using urinary proteome (naturally occurring peptides of less than 20 kDa) analysis. The use of urinary proteome analysis allows obtainment, in a non-invasive manner, of information on ageing. We have previously shown this in a number of studies which included over 1200 healthy individuals [7,8]. Comparison of human and mouse age-related urinary proteomes should provide unique insight in the translatability of mouse models of ageing.

## Materials and methods

### Mice

Mice urine samples were obtained from wild-type C57BL/6 strains (N = 89). These mice included 4 weeks (N = 13), 12 weeks (N = 15), 48 weeks (N = 28), 61 weeks (N = 5), 84 weeks (N = 13) and 96 weeks (N = 15) old mice that were purchased from Janvier Labs, France. Additionally, 61 weeks old telomerase knock-out (Terc^-/-^) mice samples (N = 5) were originally generated by injecting mTR -/- WW6 ES cells to C57BL/6 recipients and were maintained since their generation in 1997 on this C57BL/6 background by in-house breeding [9,10]. All animal experiments were conducted in accordance with the German Law for the welfare of animals and were approved by the committee from the “Regierungspräsidium Freiburg” (approval number: 35–9185.81/G-11/51).

### Humans

To compare mouse ageing with human ageing, we have used the 1227 healthy subjects previously described in a human ageing study [7]. For the definition and validation of an ageing support vector machine (SVM) classifier (see Results), a training set of 50 subjects was established by randomly selecting young and old healthy subjects within this cohort of 1227 healthy subjects. For the definition of the SVM classifier young healthy subjects were considered to be between 20–39 years (N = 25) whereas older subjects were considered over 60 years (N = 25). An independent test set was also randomly selected to validate the SVM classifier and it comprised of young (20–39; N = 20), mature (40–59; N = 20) and old healthy subjects (60 and over; N = 20). The study was performed in accordance with the ethical principles in the Declaration of Helsinki and Good Clinical Practice. All datasets were derived from previous studies and were anonymised. The study was approved by the local ethics committee (approval number: 3185–2016).

### Sample preparation and proteome analysis

The proteomic analysis based on capillary electrophoresis coupled to mass spectrometry (CE-MS) for human urine samples has already been published [7]. For mouse proteomic analysis, a 150 μl aliquot of mice urine was thawed immediately before use and diluted with 150 μl of 2 M urea, 10 mM NH_4_OH containing 0.02% SDS. To remove higher molecular mass proteins, such as albumin and immunoglobulin G, the sample was ultra-filtered using Centrisart ultracentrifugation filter devices (20 kDa MWCO; Sartorius, Goettingen, Germany) until filtrate was obtained. This filtrate was then applied onto a PD-10 desalting column (GE Healthcare, Uppsala, Sweden) equilibrated in 0.01% NH_4_OH in HPLC-grade in H_2_O (Roth, Germany) to decrease matrix effects by removing urea, electrolytes, salts, and to enrich polypeptides present. Finally, all samples were lyophilised, stored at 4°C, and suspended in HPLC-grade H_2_O shortly before capillary electrophoresis coupled to mass spectrometry (CE-MS) analyses, as described [7].

CE-MS analyses were performed using a P/ACE MDQ capillary electrophoresis system (Beckman Coulter, Fullerton, USA) on-line coupled to a microTOF MS (Bruker Daltonics, Bremen, Germany) as described previously [11,12]. The ESI sprayer (Agilent Technologies, Palo Alto, CA, USA) was grounded, and the ion spray interface potential was set between –4 and –4.5 kV. Data acquisition and MS acquisition methods were automatically controlled by the CE via contact-close-relays. Spectra were accumulated every 3 s, over a range of *m/z* 350 to 3000. Accuracy, precision, selectivity, sensitivity, reproducibility, and stability of the CE-MS measurements were demonstrated elsewhere [11].

### Peptide sequencing

For sequencing, processed urine samples were separated on a Dionex Ultimate 3000 RSLS nano flow system (Dionex, Camberly UK). A 5 ml sample was loaded onto a Dionex 5 mm C18 nano trap column at a flow rate of 5 ml/min. Elution was performed on an Acclaim PepMap 75 mm C18 nano column over 100 min. The sample was ionised in positive ion mode using a Proxeon nano spray ESI source (Thermo, Fisher Hemel UK) and analysed in an Orbitrap Velos FTMS (Thermo Finnigan, Bremen, Germany). The MS was operated in data-dependent mode to switch between MS and MS/MS acquisition and parent ions were fragmented by (high-) energy collision-induced dissociation and also electron transfer dissociation. Data files were searched against *Mus musculus* entries in the Swiss-Prot database with Proteome Discoverer version 1.2 (Thermo Fisher Scientific, Bremen) with SEQUEST spectral algorithm. No fixed modification and oxidation of methionine as variable modifications were selected. Mass error windows of 10 ppm for MS and 0.05 Da (HCD; high resolution) or 0.5 Da (CID, ETD; low resolution) for MS/MS were allowed. For further validation of obtained peptide identification, the strict correlation between peptide charge at pH 2 and CE-migration time was utilised to minimise false-positive identification rates [13]. Calculated CE migration time of the sequence candidate based on its peptide sequence (number of basic amino acids) was compared to the experimental migration time. Peptides were accepted only if they had a mass deviation below ± 80 ppm and a CE-migration time deviations below ± 2 min. Only sequenced peptides were considered for further investigation.

### Data processing

Mass spectral peaks representing identical molecules at different charge states were deconvoluted into single masses using MosaiquesVisu software [14]. Only signals with z>1 observed in a minimum of three consecutive spectra with a signal-to-noise ratio of at least four were considered. CE-MS data were calibrated using 150 reference mass data points and 452 reference migration time data points by locally weighted regression. For normalisation of analytical and urine dilution variances, signal intensities were normalised relative to 29 internal standard peptides [15]. The obtained peak lists characterise each peptide by its molecular mass [Da], normalised CE migration time [min] and normalised signal intensity. All detected peptides were deposited, matched, and annotated in a Microsoft SQL database allowing further statistical analysis [16]. For clustering, peptides in different samples were considered identical if mass deviation was <50 ppm for small (<4,000 Da) or 75 ppm for larger peptides. Acceptable migration time deviation was, < ± 1 minutes for 19 min, gradually increasing to, < ± 2.5 min at 50 min. The mass spectrometry proteomics data have been deposited to the ProteomeXchange Consortium via the PRIDE [17] partner repository with the dataset identifier PXD005229.

### Correlation analysis and development of a high dimensional model

As peptide profiles across the samples were not normally distributed, a correlation analysis was performed for peptide discovery using the non-parametric Spearman’s rank coefficient to estimate the correlation of individual peptides using age as a continuous variable. All peptides present in the full cohort were included in the correlation analysis since a frequency threshold was not set. The statistical significance was assumed at p-value less than 0.05. The p-value was adjusted by applying Benjamini-Hochberg [18] using an R-based statistical software (version 2.15.3). Generation of Box-Whisker plots and the Mann-Whitney test were performed and verified with MedCalc version 8.2.1.0 (MedCalc Software, Mariakerke, Belgium). MosaCluster (version 1.7.0) was used to build a classifier based on support vector machine (SVM) that allows the classification of samples in the high dimensional data space [19,20]. MosaCluster calculated classification scores based on the amplitudes of ageing peptides. Classification is performed by determining the Euclidian distance (defined as the SVM classification score) of the vector to a maximal margin hyperplane. The SVM-classifier uses the log transformed intensities of x features (peptides) as coordinates in an x-dimensional space. It then builds an x-1 dimensional hyperplane that spans this space by performing a quadratic programming optimisation of a Lagrangian using the training labels only while allowing for samples to lie on the wrong side of the plane. For such mistakes in classification the SVM introduces a cost parameter C. Because non separable problems in low dimensions may be separable in higher dimensions, the SVM uses the so called Kernel-trick to transform the samples to a higher dimensional space. MosaCluster uses the standard radial basis functions as kernel. These functions are just Gaussians with the parameter gamma controlling their width. There are generally implemented in SVMs in all popular data mining software, particularly the kernlab cran contributed R package is a versatile tool for building SVM based-classifiers [21]. After identification of significant biomarkers and generation of different classifiers, they were assessed in a test set or a validation set to check their performance.

### Orthology

Orthology between age-correlated mouse and human peptides was defined as follows: i) identical fragments, ii) peptides derived from the same protein region with a minimum overlap of 6 amino acids between the two species and iii) peptide sequences with a one amino acid gap [22]. In addition only peptides with the same correlation direction with age in mice and humans were retained.

## Results

### Identification of age-correlated peptides in mice

**Fig 1:**
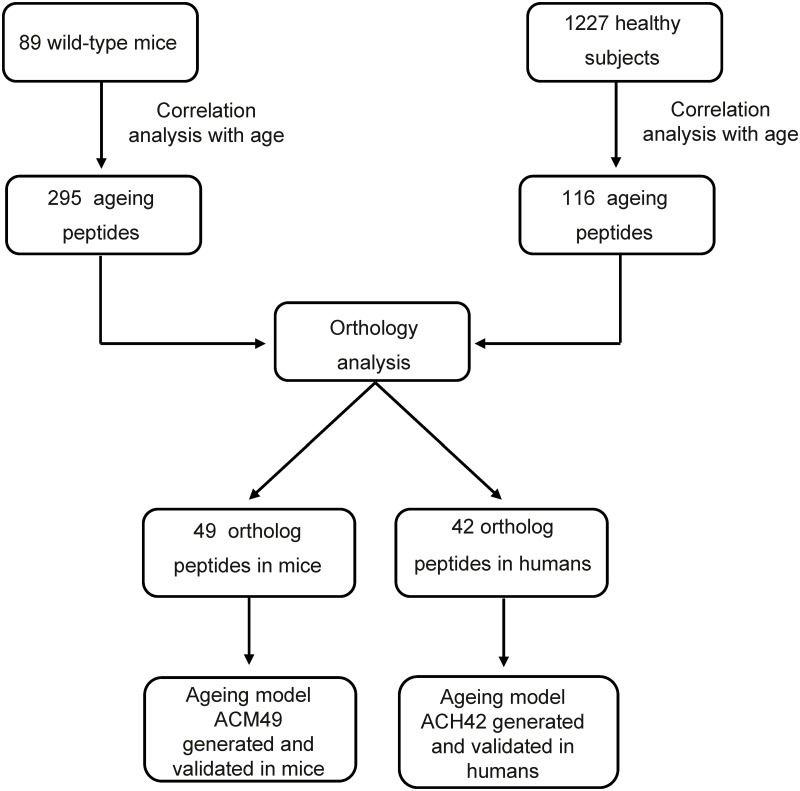
Summary of the study design. The human study has already been published [5]. The orthology analysis enabled to identify 49 ortholog peptides in mice, equivalent to 42 peptides in humans. Then ageing models were generated using ortholog peptides in mice and humans. ACM49: ageing classifier in mouse containing 49 peptides, ACH42: ageing classifier in human containing 42 peptides.

To identify peptides associated with ageing, the urinary proteome profiles of a cohort of 89 C57BL/6 wild-type mice were analysed by CE-MS (Fig 1). A correlation analysis of individual peptides with age was performed using age as a continuous variable and 295 peptides were significantly correlated with age (p≤ 0.05) (S1 Table). A considerable number of peptides (42%) associated to mouse ageing were fragments of different collagen proteins, similarly to what was observed in human ageing [7,8]. Additionally, some age-correlated peptides were only identified in mice including fragments of kidney androgen-regulated protein, complement factor D and pro-epidermal growth factor (S1 Table). Non collagen peptides were predominantly positively correlated with age and included fragments of kidney androgen-regulated protein, pro-epidermal growth factor, alpha-1-antitrypsin, major urinary protein, meprin A subunit alpha, complement factor D, uromodulin and serine protease inhibitor A3K whereas collagen fragments were predominantly negatively correlated with age and included collagen alpha-1(I) chain, collagen alpha-1(III) chain and collagen alpha-2(I) chain (S1 Table, see rho factor). The two most strongly negatively age-correlated peptides were collagen alpha-1(III) chain and collagen alpha-1(I) chain fragments (respectively ρ = -0.787, p< 0.0001 and ρ = -0.739, p< 0.0001 Table 1) whereas the two most strongly positively correlated peptides corresponded to E3 ubiquitin-protein ligase and a kidney androgen-regulated protein fragments (respectively, ρ = 0.717, p< 0.0001 and ρ = 0.709, p< 0.0001 respectively Table 1).

**Table 1:** The 20 best age-correlated peptides identified in mice.

Rho factor	Adjusted p-value	Sequence	Protein name	Start AA	Stop AA
**-0.787**	**3.08E-16**	**GSPGAKGEVGpAGSPGSNGSPGQRGEpGpQ**	**Collagen alpha-1(III) chain**	**344**	**373**
**-0.739**	**2.50E-13**	**GQpGAKGEpGDTGVKGDAGPpGP**	**Collagen alpha-1(I) chain**	**810**	**832**
**-0.680**	**9.48E-11**	**GQPGAKGEpGDTGVKGDAGPpGP**	**Collagen alpha-1(I) chain**	**810**	**832**
**-0.678**	**1.10E-10**	**ppGpAGAAGPAGNPGADGQpGAKG**	**Collagen alpha-1(I) chain**	**364**	**387**
**-0.620**	**2.14E-08**	**GLPGppGPpGEGGKQGDQ**	**Collagen alpha-1(I) chain**	**531**	**547**
**-0.582**	**3.34E-7**	**GLPGppGPpGEGGKQGDQ**	**Collagen alpha-1(II) chain**	**666**	**683**
**-0.577**	**4.5E-07**	**YKGmVGSIGAAGpPGEEGPRGppGEAG**	**Collagen alpha-2(IX) chain**	**250**	**276**
**-0.553**	**1.73E-06**	**DEAGSEAHREGETR**	**Fibrinogen alpha chain**	**527**	**540**
**-0.551**	**1.94E-06**	**KGTAGEpGKAG**	**Collagen alpha-1(I) chain**	**575**	**585**
**-05.47**	**2.51E-06**	**AGPpGPTGPTGPp**	**Collagen alpha-1(I) chain**	**320**	**332**
*0*.*543*	*2*.*98E-06*	*SLNEKLQN*	*Coiled-coil domain-containing protein 18*	*1069*	*1076*
*0*.*560*	*1*.*15E-06*	*ELQNSIIDLLNS*	*Kidney androgen-regulated protein*	*30*	*41*
*0*.*564*	*9*.*15E-7*	*VSINKELQNSII*	*Kidney androgen-regulated protein*	*25*	*36*
*0*.*572*	*5*.*99E-7*	*LVSINKELQNSIIDLLNS*	*Kidney androgen-regulated protein*	*24*	*41*
*0*.*597*	*1*.*15E-07*	*SINKELQNSIIDLLNS*	*Kidney androgen-regulated protein*	*26*	*41*
*0*.*599*	*1*.*07E-7*	*AAPEIILGNPV*	*Triple functional domain protein*	*2962*	*2972*
*0*.*694*	*2*.*54E-11*	*LVSINKELQNS*	*Kidney androgen-regulated protein*	*24*	*34*
*0*.*697*	*1*.*92E-11*	*EEHTQSPIFLGKVVDPTHK*	*Alpha-1-antitrypsin 1–1*	*395*	*413*
*0*.*709*	*6*.*35E-12*	*VSINKELQNS*	*Kidney androgen-regulated protein*	*25*	*34*
*0*.*717*	*3*.*00E-12*	*MPSLVVVSGGNSLNNLI*	*E3 ubiquitin-protein ligase HERC2*	*2846*	*2862*

In bold are negatively correlated peptides and positively correlated peptides are in italic. p = hydroxylated proline.

### Assessment of urinary proteome similarity in wild-type mice and humans ageing: Individual peptides

We have recently identified urinary peptides related to human ageing [7]. Briefly, the urinary proteome of 1227 healthy individuals between (20–86 years old) was analysed and correlated with age. A total of 116 peptides predominantly made up of different collagen fragments (72%) were found to be associated to apparent healthy human ageing [7]. Collagen fragments were predominantly negatively correlated and comprised mainly collagen alpha-1(I) chain, collagen alpha-1(III) chain, and collagen alpha-2(I) chain whereas non collagen fragments were predominantly positively correlated and comprised for example fibrinogen fragments and uromodulin.

**Fig 2:**
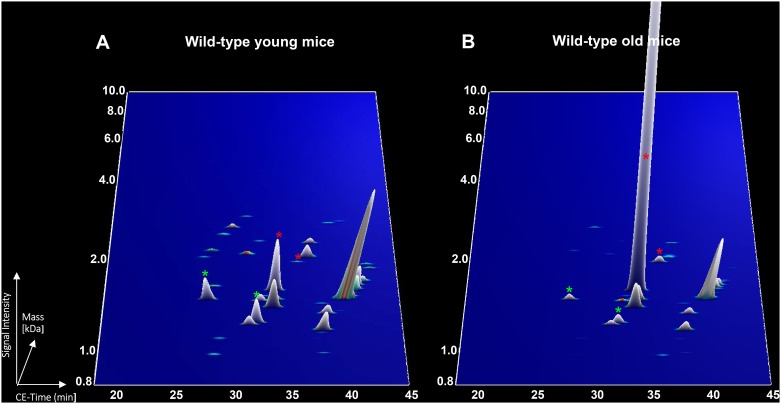
Abundance of ortholog peptides in wild-type mice. (A) Young mice (4 weeks old) and (B) wild-type old mice (84 weeks old). The green stars represent collagen alpha-1(I) chain peptides where as the red stars represent uromodulin peptides.

**Fig 3:**
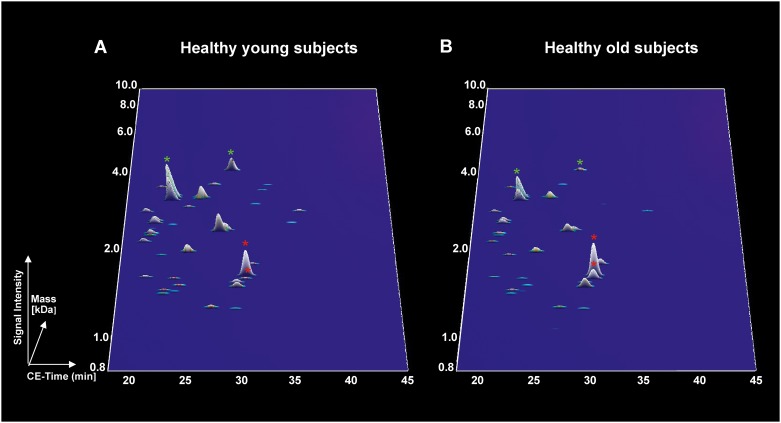
Abundance of ortholog peptides in healthy subjects. (A) Young subjects (20–39 years old) and (B) healthy old subjects in (60 years old and over). The green stars represent collagen alpha-1(I) chain peptides where as the red stars represent uromodulin peptides.

Since collagen fragments and uromodulin fragments were observed to be associated to both mouse and human ageing and represented the majority of the peptides, they were further used to evaluate the similarity between mouse and human ageing based on orthology analysis. Forty nine unique sequences in mice showed orthology to 42 unique sequences in humans, although 1 peptide in mice could have several corresponding ortholog peptides in humans and *vice versa* (Table 2, S2 Table). Ortholog sequences included collagen alpha-1(I) chain, collagen alpha-1(III) chain, collagen alpha-2(I) chain and uromodulin. Proteome profiles of the ortholog peptides in young and old wild-type mice and healthy subjects were depicted for visual representation. The decreased of collagen alpha-1(I) (respectively Figs 2 and 3, green stars) and the increased of uromodulin peptides (respectively Figs 2 and 3, red stars) with age were depicted. Overall, of the 295 peptides associated to mouse ageing, 49 displayed similarity with urinary peptides of human ageing in healthy individuals.

**Table 2:** Ortholog peptides identified in mice and humans.

Peptide ID mice	Sequence mice	Peptide ID humans	Sequence humans	Gene symbol
1504650	**GPpGKNGDDGEAGKpGRp**GERGPpGP	68411	Pp**GPpGKNGDDGEAGKpGRp**	COL1A1
16415	**KNGDDGEAGKpG**RpG	41476	GPPG**kNGDDGEAGKPG**	COL1A1
1504057	**KNGDDGEAGKpGRpGERGPpGP**^**1**^	102819	GPpG**KNGDDGEAGKPGRpGERGPpGp**Q	COL1A1
1504810	**KNGDDGEAGKpG**RPGERGpPGP	38011	PpG**KNGDDGEAGKpG**	COL1A1
19556	**GEAGKPGRpGERGPpGP**^**2**^	93897	PpGKNGDD**GEAGKPGRpGERGppGP**	COL1A1
19556	**GEAGKPGRpGERGPpGP**^**2**^	99919	PpGKNGDD**GEAGKPGRpGERGppGP**Q	COL1A1
6931	**GRpGERGPpGP**	87365	KNGDDGEAGKp**GRpGERGPPGp**Q	COL1A1
1504057	**KNGDDGEAGKpGRpGERGPpGP**^**1**^	89642	**KNGDDGEAGKPGRpGERGPPGp**QG	COL1A1
7545	**AGPpGPTGP**T**GPp**^**3**^	141007	ARGNDGATGA**AGPpGPTGP**A**Gpp**GFpGAVGAKGEAGPQGPRG	COL1A1
7545	**AGPpGPTGP**T**GPp**^**3**^	155132	ARGNDGATGA**AGpPGPTGP**A**GPP**GFpGAVGAKGEAGpQGpRGSEGPQG	COL1A1
37057	NSGEpG**ApG**N**KGDTGAKGEpG**ATGVQGPpGP	50172	**ApG**S**KGDTGAKGEpG**PVG	COL1A1
18625	TG**SpGSPGPDGKTGPp**GP	29538	**SpGSPGPDGKTGPp**	COL1A1
18875	TG**SpGSpGPDGKTGPpGP**	46649	**SpGSPGPDGKTGPpGP**AG^7^	COL1A1
20909	TG**SpGSPGPDGKTGPpGPAG**	46649	**SpGSPGPDGKTGPpGPAG**^**7**^	COL1A1
16583	G**SpGSPGPDGKTGPpGP**	29538	**SpGSPGPDGKTGPp**^**8**^	COL1A1
16886	G**SpGSpGPDGKTGPp**GP	29538	**SpGSPGPDGKTGPp**^**8**^	COL1A1
19345	G**SpGSpGPDGKTGPpGPAG**	46649	**SpGSPGPDGKTGPpGPAG**^**7**^	COL1A1
18066	**SpGSPGPDGKTGPpGPAG**	46649	**SpGSPGPDGKTGPpGPAG**^**7**^	COL1A1
3902	**KG**T**AGEpGKAG**	117371	VMGFPGp**KG**A**AGEPGKAG**ERGVpGppGAVGPAG	COL1A1
1506170	T**AGEpGKAGERG**L**pGPpG**^**4**^	122825	**GEpGKAGERG**V**pGPPG**AVGpAGKDGEAGAQGPPGP	COL1A1
1506170	T**AGEpGKAGERG**L**pGPpG**^**4**^	127351	A**AGEPGkAGERG**V**pGPpG**AVGPAGKDGEAGAQGPPGP	COL1A1
1505741	**ERGEQGPAGSpG**	135166	**ERGEQGPAGSpG**FQGLpGPAGPpGEAGKpGEQGVPGD	COL1A1
19814	**GLPGpAGPpGEAGKpGEQ**	100255	**GLPGpAGppGEAGKPGEQ**GVPGDLGApGP^9^	COL1A1
4170	**KpGEQGVPGD**	100255	GLPGpAGppGEAG**KPGEQGVPGD**LGApGP^9^	COL1A1
17232	**KpGEQGVpGDLGApGP**	100255	GLPGpAGppGEAG**KPGEQGVPGDLGApGP**^9^	COL1A1
16079	**GQPGAKGEpGD**TGVKG	92841	AD**GQPGAKGEpGD**AGAKGDAGPpGPAGP^10^	COL1A1
26939	**GQPGAKGEpGD**TGVKGDAGPpGP	121241	AD**GQPGAKGEpGD**AGAKGDAGPpGPAGPAGPPGPIG	COL1A1
27161	G**QpGAKGEpGD**TGVKGDAGPpGP	28850	DG**QPGAKGEpGD**AG	COL1A1
27388	G**QpGAKGEpGD**TGVKGDAGppGP	40344	DG**QPGAKGEPGD**AGAK	COL1A1
9877	**PGAKGEpGD**TGVK	44802	DGQ**PGAKGEpGD**AGAKG	COL1A1
14420	**PGAKGEpGD**TGVKGD	92841	ADGQ**PGAKGEpGD**AGAKGDAGPpGPAGP^10^	COL1A1
24239	**ETGPAGRpGEVGPpGPpGPAG**	140803	**ETGPAGRpGEVGPpGpPGPAG**EKGSPGADGPAGAPGTPGPQG^11^	COL1A1
20667	**GEVGPpGPpGpAGEKGSpG**	140803	ETGPAGRp**GEVGPpGpPGPAGEKGSPG**ADGPAGAPGTPGPQG^11^	COL1A1
38376	**ESGREG**S**pGAEGSpGRDG**A**pGAKGDRGETGP**	127432	GPpG**ESGREG**A**PGAEGSpGRDG**S**pGAKGDRGETGp**	COL1A1
35125	**REG**S**pGAEGSpGRDG**A**pGAKGDRGETGP**^**5**^	130077	GPpGESG**REG**A**pGAEGSpGRDG**S**pGAKGDRGETGP**A	COL1A1
35125	REGSpGA**EGSpGRDG**A**pGAKGDRG**ETGP^5^	51175	**EGSpGRDG**S**pGAKGDRG**	COL1A1
10361	DGAp**GAKGDRGET**	124886	PpGESGREGAPGAEGSpGRDGSp**GAKGDRGET**GP	COL1A1
17249	**pGPVGPAGK**N**GDRGET**	128435	DRGETGPAGPpGApGAPGA**PGPVGpAGK**S**GDRGET**GP	COL1A1
25169	QGI**pGTGGPpGENGKpGEpGP**	16910	GL**pGTGGPpGENGKPGEPGp**	COL3A1
15377	GI**pGTGGPpGENGKpG**	61304	GL**pGTGGPpGENGKpG**EPGpKG^12^	COL3A1
12858	**GPpGENGKpGEpGP**	61304	GLpGTG**GPpGENGKpGEPGp**KG^12^	COL3A1
31210	Q**NGEpG**A**KGERGApGEKGEGGPpG**P	70911	GAPGQ**NGEPG**G**kGERGApGEKGEGGPpG**	COL3A1
27744	GEpGAKGERG**ApGEKGEGGPpGP**	97638	**ApGEKGEGGPpG**^**13**^	COL3A1
8738	**ApGEKGEGGPpG**P	97638	**ApGEKGEGGPpG**^**13**^	COL3A1
1505438	**ApGLpGPRGIpGP**AG	126318	TGAkGAAGLpGVAG**ApGLpGPRGIpGP**VGAAGATGARG	COL1A2
1506234	KGEQ**GpAGP**P**G**FQGLpG	138279	EVGKpGERGLHGEFGLp**GpAGp**R**G**ERGPPGESGAAGP	COL1A2
30714	SGTTG**EVGKpGERGL**p**GEFGLpGP**	148645	**EVGKpGERGL**H**GEFGLPGP**AGpRGERGpPGESGAAGPTGPIG	COL1A2
27017	**DGPpGRDGQpGHKGERGYpG**^**6**^	112515	GRDGNpGN**DGPpGRDGQpGHKGERGYpG**	COL1A2
27017	**DGPpGRDGQpGHKGERGYpG**^**6**^	80306	N**DGPpGRDGQpGHKGERGYpG**	COL1A2
36597	PGKDGEV**GPSGPVGPPGLAGERGEQGppGP**	113351	**GpSGpVGpPGLAGERGEQGPpGP**TGFQGLPG	COL5A2
25768	SGNF**IDQ**T**RVLNLGPI**TR	53181	SGSV**IDQ**S**RVLNLGPI**	UMOD
6864	F**IDQ**T**RVLN**	43605	SV**IDQ**S**RVLN**LGPI	UMOD
18643	I**DQ**T**RVLNLGPIT**R	50056	SV**IDQ**S**RVLNLGPIT**^**14**^	UMOD
16362	**DQ**T**RVLNLGPIT**R	50056	SVI**DQ**S**RVLNLGPIT**^**14**^	UMOD
3736	**VLNLGPIT**R	50056	SVIDQSR**VLNLGPIT**^**14**^	UMOD

The superscripted numbers represent sequences that have several ortholog in either mice or humans. In bold are the ortholog sequences. p = hydroxylated proline.

### Assessment of urinary proteome similarity in wild-type mice and humans ageing: Use of multidimensional peptide models

**Fig 4:**
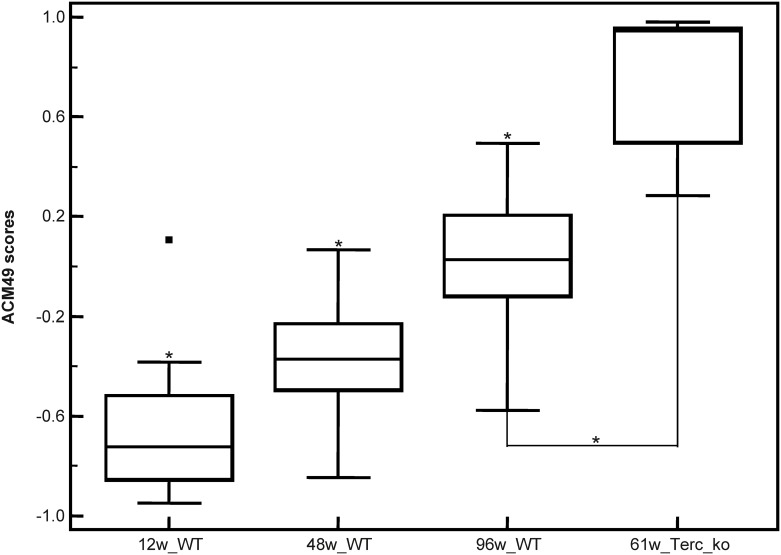
Box-whisker plot depicting the age classification in wild-type and telomerase knock-out mice. * Statistically significant p-value, WT: wild-type, Terc^-/-^: telomerase knock-out mice and w: weeks.

To further investigate whether the mouse urinary peptides were representative of human ageing, we developed multidimensional classifiers based on the ortholog peptides. We scored mice and human age using these classifiers based on the hypothesis that correct age classification by these ortholog peptides in both mice and humans validates the translatability of the mouse peptides. With this aim, an ageing classifier was developed, using the 49 ortholog age-correlated mouse peptides, along with a training cohort of wild-type mice (N = 39) using support vector machine (SVM)-based modelling. This resulted in the classifier called ACM49 (ACM: ageing classifier in mouse). To validate the classifier, a cohort of 45 wild-type mice including young (12 weeks; N = 15), mature (48 weeks; N = 15), and old (96 weeks; N = 15) were used. In this validation, the ACM49 classifier was able to discriminate between the different age groups with p< 0.0001 in young versus old; p = 0.0030 in young versus mature; and p = 0.0045 in mature versus old. As depicted in the Box-Whisker plot (Fig 4), the median scores from the ACM49 classifier increased proportionally to the age of wild-type mice. Additionally, ACM49 was also used to assess the age of telomerase knock out mice samples (N = 5). Findings revealed that classification scores of 61 weeks old telomerase knock out mice were significantly higher (p = 0.0019) than of 96 weeks old wild-type mice (Fig 4).

**Fig 5:**
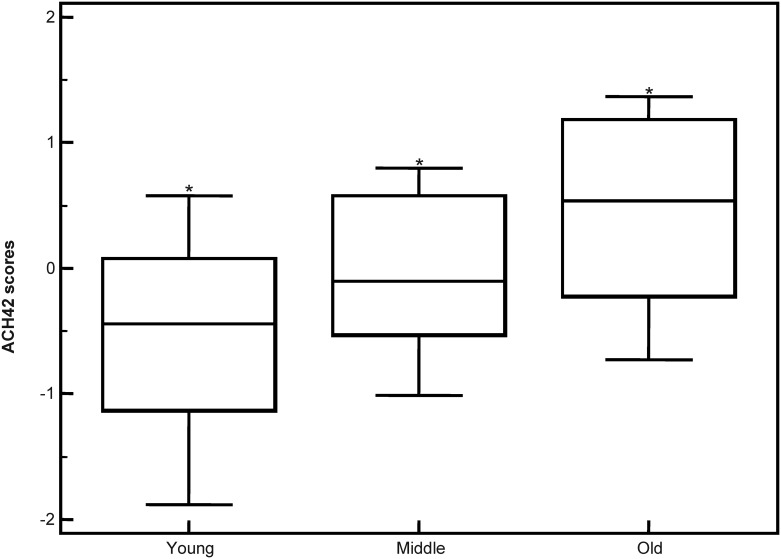
Box-whisker plot depicting the age classification in healthy subjects. Young subjects are between 20–39 years, mature subjects are between 40–59 years and old subjects are 60 years old and over. * Statistically significant p-value.

The same peptides, but now ortholog in humans, comprised of 42 peptides (S2 Table) were modelled in a classifier called ACH42 (ACH: ageing classifier in human) using a training human cohort of healthy subjects (N = 50). To validate the classifier, an independent cohort of 60 healthy subjects including young (20–39 years; N = 20), mature (40–59 years; N = 20) and old (60 years and over; N = 20) were used. In this independent validation the ACH42 classifier was able to discriminate between the different age groups with p = 0.0005 in young vs old; p< 0.0001 in young vs mature; and p = 0.0453 in mature vs old. The classifier was significantly able to discriminate between different age groups as younger subjects had a lower median score generated by the ACH42 classifier which progressively increased with age (Fig 5). The data suggested that urinary peptide classifiers exclusively comprising ortholog peptides, can discriminate the age in both species.

## Discussion

The urinary proteome profiles of 89 wild-type mice were analysed and compared with the proteome profiles of a unique cohort of 1227 healthy subjects to evaluate the translatability of ageing findings from mice to humans.

Using urinary proteomics, common molecular mechanisms describing ageing in human and mouse were identified. The difference in the number of identified peptides (295 versus 116) between mice and humans most likely lies in the high genetic variability in humans and their exposure to environmental changes leading to age-dependent changes being obstructed. Therefore, the great biological variance resulted in a lower number of biomarkers. In both species, a decrease in collagen fragments and increase in uromodulin peptides were identified as key molecular changes observed during ageing. These ortholog peptides which predominantly included collagens fragments were able to discriminate among different age groups in both human and mouse cohorts. These findings not only highlight the translatability from mice to humans, but also specifically point out that this translatability is mostly attributed to collagens. Thus, findings highlight the key role of collagens in human and mouse ageing processes.

Indeed, the pivotal role of collagens in ageing has previously been shown [3,7,8]. However, the strong homology in mouse and human collagens in ageing via investigation of the urinary proteome has not been shown before. This observation suggests that mice can be used to assess anti-ageing interventions targeting collagens [23] in humans. As a disturbance in the collagen homeostasis is associated with several chronic age-related conditions including cancers [24], chronic kidney diseases (CKD) [25] and cardiovascular diseases [26], mice can also be used to develop therapeutic interventions for humans against these age-associated pathologies.

Furthermore, the identification of age-correlated ortholog peptides enables to gain increased insight into molecular mechanisms involved during human and mouse ageing. In humans, ageing was characterised by the decrease of fibrillary collagen fragments, especially collagen alpha-1(I) chain, collagen alpha-1(III) chain and collagen alpha-2(I) chain. The reduction in collagen type I and type III synthesis has previously been associated to skin ageing [27] and to systemic ageing [7,8]. Indeed, a reduction in collagen synthesis may indicate a perturbation in the extracellular matrix (ECM) remodelling. The ECM is ubiquitous in the organism and plays a pivotal role in tissue elasticity and integrity [28]. Perturbations of the ECM have been associated with several pathologies [28]. For instance, perturbations in the ECM have been shown to cause fibrosis; a condition characterised by an abnormal accumulation of ECM components. Fibrosis has been associated with renal diseases, cardiovascular diseases and cancers [29]. Hence in humans, ageing is characterised by a perturbation in collagen homeostasis which can lead to fibrosis formation followed by a myriad of age-related complications.

In mice, the urinary excretion of fibrillar type I and III collagen fragments representing the majority of collagenous peptides decreased with increasing age. This may be reflective of increased cross-linking and subsequently increased collagen biosynthesis and decreased activity of matrix metalloproteinases and other collagen degrading enzymes [30]. In a recent study, a mouse model developing resistance to type I collagenase activity (Col1a1^r/r^) was demonstrated to promote premature ageing [31]. The Col1a1^r/r^ mice were shown to have shortened lifespan in comparison to WT mice and they developed hypertension caused by aortic stiffness. Interestingly, collagenase-resistant collagen was reported to promote premature ageing by inducing senescence in vascular smooth muscle cells due to inadequate communication with αvβ3 integrin [31]. Moreover, a type III collagen-deficient mouse was generated to assess bladder function [32]. It was shown in this study that deficiency in type III collagen fragment caused reduced tension or elasticity subsequently resulting in impaired bladder contraction and development [32]. In humans, resistance in collagen degradation by collagenases has been previously shown to predict the chronological age using diaphragm tissues obtained from biopsies [33]. In addition to collagen fragments, uromodulin was shown to increase with age. Uromodulin is a protein exclusively produced in the kidney and the most abundant protein in urine. Its biological function still remains unknown however; deregulation in its synthesis has been reported to be associated with hypertension and chronic kidney diseases [34].

Ageing classifiers were established based on ortholog peptides and these classifiers could discriminate the age in both humans and wild-type mice. Hence, these findings demonstrate the translatability of results from mouse to human based on urinary proteome analysis. Similar observations were also made while using urinary proteome analysis to compare between rat and human in a previous study [35]. It was observed that the ZDF rat model for type 2 diabetes mellitus displayed similarity to human cardiovascular diseases rather than chronic kidney diseases based on ortholog peptides [35]. Furthermore, the mouse ageing classifier ACM49 was able to discriminate the age in wild-type mice and also the internal age in telomerase knock-out (Terc^-/-^) mouse models characterised by a short lifespan. The ACM49 revealed a discrepancy between the chronological age of Terc^-/-^ and their internal age as the 61 weeks old Terc^-/-^ mouse models had higher scores compared to 96 weeks WT mice. Though findings in mice are translatable in humans when focusing on ortholog peptides, this observation cannot be generalised to all identified age-related peptides. Indeed looking at the differences in the number of identified peptides, and also looking at the unique peptides specific to each species, it is obvious that findings using mouse models must be interpreted with care.

In conclusion, we have demonstrated that mice can be good models to study human ageing when focusing on ortholog peptides. One major advantage of using urinary proteomics to study ageing, as suggested by the results, is the ability to obtain a representative readout of human ageing using mouse models, hence, allowing eventually to investigate interventions in the management of ageing-associated complications.

## Supporting information

S1 Table Age-correlated peptides identified in wild-type mice. 1 means human, 2 means mouse and 1, 2 means human and mouse.
(XLS)

S2 Table Ortholog peptides identified in mice and humans. The superscripted numbers represent sequences that have several ortholog in either mice or humans. In bold are the ortholog sequences.
(XLS)
